# Reply to Huerta et al. Comment on “Larroya et al. Validity and Reproducibility of a Spanish EPIC Food Frequency Questionnaire in Children and Adolescents. *Nutrients* 2024, *16*, 3809”

**DOI:** 10.3390/nu18091423

**Published:** 2026-04-30

**Authors:** Ana Larroya, María Tamayo, María Carmen Cenit, Yolanda Sanz

**Affiliations:** Microbiome, Nutrition & Health Research Group, Institute of Agrochemistry and Food Technology, Spanish National Research Council (IATA-CSIC), 46980 Valencia, Spain

First of all, we would like to express our gratitude for your careful reading of our work and acknowledge the constructive nature of the observations [1]. We welcome this opportunity to clarify certain points that may have led to confusion and to provide the necessary context to ensure an accurate interpretation of our study [2]. Moreover, we apologize for any misunderstandings regarding the methodology and justification.

## 1. On the Origin and Naming of the FFQ Used

We acknowledge the concern raised regarding the terminology “Spanish EPIC FFQ” used in the title and manuscript. The food frequency questionnaire (FFQ) applied in our study was based on the 130-item semi-quantitative questionnaire originally developed within the EPIC-Norfolk study in the United Kingdom [3], and not on dietary instruments used within the EPIC-Spain cohorts. The version we used was translated into Spanish and adapted to reflect local dietary patterns, resulting in a 137-item FFQ appropriate for use in the Spanish infant and adolescent population. Therefore, our study represents the first adaptation and validation of the EPIC-Norfolk FFQ in a Spanish population.

We agree that referring to the FFQ as a “Spanish EPIC FFQ” may have led to the mistaken impression that this tool had previously been used in or validated for Spanish adult populations or that it derived from the EPIC-Spain study. We apologize for this imprecision and confirm that the FFQ used is not related to the diet history questionnaire employed by EPIC-Spain centers.

## 2. Clarification on References and Prior Validation

In the original manuscript, reference 15 [4] was incorrectly cited as evidence of the validation of the FFQ in Spanish adults. We appreciate the EPIC-Spain group for bringing this error to our attention. We have revised our reference list accordingly and note that, to the best of our knowledge, the EPIC-Norfolk FFQ has not previously been validated in a Spanish population, either adult or pediatric, although it has been validated in other countries [5,6]. Therefore, our study represents the first attempt to adapt and validate this particular dietary assessment tool for use in Spanish children and adolescents.

## 3. Title and Language Amendments

In light of these observations, we suggest that the title of the article may be more appropriately phrased as: “Validity and Reproducibility of an Adapted Version of the EPIC-Norfolk Food Frequency Questionnaire in Spanish Children and Adolescents.” We understand that changes to the published title may not be feasible, but we agree that this title may more accurately reflect the scope and origin of the FFQ used.

## 4. Scientific Collaboration

We would like to note that in September 2022 we contacted the EPIC group in Murcia to inquire about dietary assessment tools. We were told that the methodology used in the EPIC-Spain cohort was the Dietary History, validated in adults but not in children [7,8,9]. In that message, we mistakenly understood that by ‘Dietary History’ they meant the FFQ-Norfolk. In this regard, we would like to apologize again for the misunderstanding.

## 5. Conclusions

We appreciate the EPIC-Spain group for their engagement with our work and for helping to clarify these important points regarding the provenance and adaptation of dietary assessment tools. We believe that these clarifications will enhance the understanding and interpretation of our study and contribute to the ongoing development of validated tools for dietary assessment in nutritional studies for pediatric populations.

Finally, we also want to emphasize that the original article represents the first assessment of this instrument in a Spanish population and the results remain valid and significant. The methodology followed recognized guidelines for dietary assessment validation, including established recommendations regarding sample size, selection of reference methods, seasonal coverage, statistical correction for within-person variability, energy adjustment, de-attenuation procedures, and multiple agreement analyses. Thus, by adhering to these widely accepted methodological standards, the present study provides an additional transparent and reproducible validation strategy that can be applied in other cultural and geographical contexts.

## References

[1] Huerta J.M., Chirlaque M.D., Amiano P., Guevara M., Sánchez M.J., Agudo A. (2026). Comment on Larroya et al. Validity and Reproducibility of a Spanish EPIC Food Frequency Questionnaire in Children and Adolescents. *Nutrients* 2024, *16*, 3809. Nutrients.

[2] Larroya A., Tamayo M., Cenit M.C., Sanz Y. (2024). Validity and Reproducibility of a Spanish EPIC Food Frequency Questionnaire in Children and Adolescents. Nutrients.

[3] Bingham S.A., Welch A.A., McTaggart A., Mulligan A.A., Runswick S.A., Luben R., Oakes S., Khaw K.T., Wareham N., Day N.E. (2001). Nutritional Methods in the European Prospective Investigation of Cancer in Norfolk. Public Health Nutr..

[4] Kaaks R., Riboli E. (1997). Validation and Calibration of Dietary Intake Measurements in the EPIC Project: Methodological Considerations. Eur. Prospect. Investig. Into Cancer Nutr. Int. J. Epidemiol..

[5] Ocké M.C., Bueno-de-Mesquita H.B., Goddijn H.E., Jansen A., Pols M.A., van Staveren W.A., Kromhout D. (1997). The Dutch EPIC Food Frequency Questionnaire. I. Description of the Questionnaire, and Relative Validity and Reproducibility for Food Groups. Int. J. Epidemiol..

[6] Shatylo S., Solovyova G. (2024). Adaptation and Validation of the EPIC-Norfolk Food Frequency Questionnaire for Assessing Dietary Intake in Ukrainian Adults. BMJ Nutr. Prev. Health.

[7] (1997). Relative Validity and Reproducibility of a Diet History Questionnaire in Spain. I. Foods. EPIC Group of Spain. European Prospective Investigation into Cancer and Nutrition. Int. J. Epidemiol..

[8] (1997). Relative Validity and Reproducibility of a Diet History Questionnaire in Spain. II. Nutrients. EPIC Group of Spain. European Prospective Investigation into Cancer and Nutrition. Int. J. Epidemiol..

[9] (1997). Relative Validity and Reproducibility of a Diet History Questionnaire in Spain. III. Biochemical Markers. EPIC Group of Spain. European Prospective Investigation into Cancer and Nutrition. Int. J. Epidemiol..

